# Editorial: The next phase in heritage language studies: methodological considerations and advancements

**DOI:** 10.3389/fpsyg.2024.1392474

**Published:** 2024-03-19

**Authors:** Fatih Bayram, Maki Kubota, Sergio Miguel Pereira Soares

**Affiliations:** ^1^UiT The Arctic University of Norway, Tromsø, Norway; ^2^Max Planck Institute for Psycholinguistics, Nijmegen, Netherlands

**Keywords:** heritage language speakers, bilingualism, online methods, language processing, language maintenance

Over the past three decades, research on heritage language (HL) bilingualism has undergone significant advancement revealing the intricate dynamics of linguistic competencies among heritage speakers (HSs). As a unique subgroup within the bilingual community, these individuals typically acquire their native language(s) in environments where it is not the dominant language, often due to migration, where HLs may be spoken at home but not formally taught or reinforced in dominant societal/educational settings (e.g., Rothman, 2009; Montrul, 2016; Polinsky, 2018). Despite being native speakers of their home language(s), HSs exhibit vast outcomes variation of linguistic competence/performance compared to other bilinguals and monolingual peers (see Kupisch and Rothman, 2018). This variability has prompted researchers to explore methodologies that capture the nuances of HS linguistic knowledge and processing. This line of investigations has delved into how HSs maintain, adapt or even lose competence in their native language over time, and also explored the sociolinguistic and experiential factors that shape such observations. Traditionally, these studies were rooted in adjacent fields such as L1 acquisition and adult L2 acquisition, predominantly employing behavioral methodologies to understand HS performance. While informative, these approaches often overlooked the methodological complexities inherent in studying HS linguistic realities, which can dynamically shift across the lifespan (Bayram et al., 2021).

Recent advancements, however, have marked a paradigm shift in HL bilingualism research, with a focus on methodological innovations aimed at more accurately capturing the linguistic competencies of HSs (Bayram et al., 2021). This movement unfolds on three main fronts. Firstly, there is a departure from traditional HSs vs. non-HSs comparisons, as researchers now explore comparisons among different HS groups, seeking to comprehend HL grammars in their own right. This shift allows for a more nuanced understanding of the variations underlying HL competence. Secondly, studies have delved into the multidimensional relationship between HSs' sociolinguistic networks and their linguistic competence, acknowledging the role of individual differences within HS groups. This approach recognizes that linguistic competence is not solely shaped by exposure to the HL but is also affected by the sociolinguistic environments in which HSs are placed in. Finally, the adoption of novel (for the field) online/processing methodologies, such as eye-tracking and electroencephalography/event-related potentials (EEG/ERPs), represents another frontier. These innovative techniques provide insights into automatic language processing, offering a more granular understanding of the underlying cognitive mechanisms at play in HL competence. By employing these advanced methods, researchers aim to circumvent confounding variables that can be more challenging to tease apart in more traditional methodologies and capture a more accurate representation of the interplay between linguistic competence and processing in HSs (e.g., Pereira Soares, 2022).

By leveraging on all these innovations, the studies within this Research Topic aimed to chart the multifaceted landscape of HL bilingualism, the underlying mental systems of HL grammatical outcomes, processing, and maintenance within the context of diverse linguistic and socio-cultural environments. Drawing from a range of innovative methodologies and approaches, including offline experimental studies, psycho-/neurolinguistic studies employing online methods, and corpus analyses, the articles in this Research Topic span over a rich array of inquiry. By exploring the influence of linguistic exposure, proficiency levels, language attitudes, and socio-cultural contexts on HL competence/performance, these articles provide valuable insights into mechanisms underlying heritage language development. More importantly, they collectively contribute to the evolving landscape of HL bilingualism research, thus bridging the current state-of-the-art with future directions in HL studies.

In the three following sub-chapters, we present a comprehensive exploration of HL bilingualism, highlighting the methodological intricacies and theoretical implications that shape the current understanding of this complex linguistic phenomenon.

The first group of studies focus on assessing individual experiences and HL competence/performance via employment of detailed questionnaires and/or other background measures. Tomić et al.'s validation of the Heritage Language Experience (HeLEx) questionnaire provides a comprehensive assessment tool for documenting heritage bilingualism, highlighting the importance of methodological choices in assessing language background and proficiency levels. They proposed a comprehensive online questionnaire for documenting heritage bilingualism, validated against an extended version of an already existing questionnaire, revealing important distributional patterns in their data. In a similar vein, Perez-Cortes and Giancaspro's exploration of frequency effects in HL acquisition underscores the complexity of linguistic development among bilingual individuals, emphasizing the need for comprehensive (subjective) assessments of language exposure and proficiency. Similarly, Macbeth et al.'s study on bilingual language experiences underscores the importance of employing diverse assessment methods to capture the intricacies of real-world language use among HSs. They examined bilinguals' language experiences using self-report questionnaires and audio recordings, revealing significant predictors of real-world language use via self-reported language use and age of English acquisition. van Osch et al. examined adjective-noun word order in code-switching among Spanish and Papiamento HSs in the Netherlands. They found that both linguistic (e.g., matrix, type of insertion) and non-linguistic (e.g., age, exposure, use) aspects influence how HSs navigate code-switching, and that children may require more time or exposure for adult-like norms. Focusing on the linearization of constituents at the right sentence periphery in German, Tsehaye's study analyzed spoken and written productions from English-German HSs and monolingually-raised speakers of German in different registers. Their findings offer insights into the impact of language contact and exposure on syntactic variation, contributing to our understanding of language change and adaptation. Assessing a different syntactic domain, Arechabaleta Regulez and Montrul's analysis of differential object marking (DOM) among Spanish HSs and L2 learners also found that type of task and type of sentence each have an effect on speakers' use of DOM, together with experiential factors such as language experience and practices. Finally, Kutlu et al.'s research on speech perception among bilingual communities introduces a novel approach to examining categorical perception, challenging existing theories and highlighting the need for a more precise understanding of speech categorization. They reexamined the theory of categorical perception in speech, introducing the Visual Analog Scaling task to enable a more precise examination of speech categorization in diverse bilingual communities, specifically HSs who often show gradient speech perception across different contexts.

The focus of the next cohort of studies is understanding the impact of socio-economic, cultural, and educational factors on the multifaceted and diverse nature of HLs. Firstly, Nguyen et al.'s study draws attention to the socio-economic factors influencing language course enrollment and performance among HSs, shedding light on disparities in educational access and outcomes within bilingual communities, e.g., by highlighting the impact of disability status, poverty, and prior academic performance. By examining and emphasizing language proficiency and cultural identity among heritage speakers, Hayakawa et al.'s work uncovered the predictors of language proficiency, vocabulary, and cultural identification in different groups of HSs, highlighting the importance of accounting for individual language history (such as overall HL exposure, HL experience in informal and formal contexts). The next two studies draw attention to diverse aspects of immigration influence on HLs. Wang et al.'s cross-sectional exploration of emotional experiences within Chinese and African immigrant families underscores the significance of language emotions in shaping family language policies and language ideologies, providing valuable insights into the socio-emotional dimensions of HL maintenance. Antonova-Unlu and Bayram's investigation into HL performance among Turkish-German returnees (into Turkey) sheds light on the challenges and opportunities faced by individuals reintegrating into their HL community, highlighting the role of external factors (the length of residence, the age at return to the homeland, and the frequency of HL use in the migration context) in language proficiency, maintenance and (re-)activation of their HL. Finally, Bar On and Meir's investigation into speech act pragmatics among HSs sheds light on the cross-cultural and cross-linguistic differences in request and apology realizations. They compared English (HL)-Hebrew adult speakers in Israel with Hebrew-dominant and English-dominant speakers. They found distinct hybrid strategies in requests and apologies among HSs, showing cross-cultural and cross-linguistic differences in their pragmatic competencies.

The third and last set of studies employed a diverse array of psycho-/neurolinguistic methods to understand how HL processing unfolds in the minds of HSs. Uygun examined the real-time sentence processing of plural-marked and unmarked verbs in sentences with overt and null subjects using self-paced reading task (SPRT) among Turkish HSs. Their results show both qualitative and quantitative differences in processing strategies between Turkish HSs and Turkish non-HSs, suggesting that Turkish HSs do indeed have the syntactic structure but may need more time to integrate this information during real-time processing, Tokaç-Scheffer et al. also used a SPRT among Turkish HSs to examine their processing of evidentiality, i.e., the linguistic marking of information source. Their findings reveal quantitative differences between HSs and non-HSs in the sense that HSs were generally slower and less accurate than non-HSs in both reading times and acceptability judgements, but both groups showed similar patterns regarding reading times on evidential-marked verb forms that matched or mismatched to the information source. The studies by Uygun and Tokaç-Scheffer et al. collectively demonstrate that when tested in both online and offline modes, HSs consistently show quantitative differences in an online paradigm, suggesting that HSs have difficulties in dealing with cognitive load that comes with real-time processing of linguistic structures. Indeed, Di Pisa et al.'s investigation of grammatical gender variability in Italian HSs show converging evidence showing that, only in an SPR paradigm, HSs show greater sensitivity to markedness (agreement violations realized on feminine adjectives) compared to non-HSs, while both groups make use of markedness information in offline grammaticality judgement task. Jegerski and Keating's study on Spanish verb argument specifications adds to the findings of other studies in this Research Topic employing SPRT, by demonstrating that lower self-ratings for reading skill in Spanish and slower average reading speed correlated to a larger spillover effect of transitivity among HSs. Their study underlines the role that general reading skills play when testing morphosyntactic processing among HSs using an online processing paradigm such as SPRT. Bentea and Marinis extends aforementioned studies using SPRT to child bilingualism, examining online comprehension and production of multiple interrogatives in Romanian-English HS children. In contrast to the findings in the adult HS literature, they found no differences in online comprehension between HS children and monolingual children, but rather significant differences emerged in production, in which HS children produced less complex wh-movement structures. Together, the studies in this Research Topic employing SPRT reveal the importance of utilizing both online and offline measures to gauge on *what* HSs know and *how* they use that knowledge in real-time linguistic processing.

While self-paced reading task is an accessible, resource-efficient method that can be used to reveal how HSs process grammatical information in real-time, the following five studies take advantage of even more granular methodologies such as eye-tracking or EEG/ERP to examine linguistic processing in HSs. Özsoy et al. addressed the predictive use of case-marking in Turkish HSs and monolinguals, using both in-lab and web-cam based eye tracking. While both groups used case-marking to predict the upcoming noun with in-lab eye tracking experiments, they were only able to replicate these results using web-based eye tracking with monolinguals, but not with HSs due to the greater variability in data collection environment. Similarly, but in a lab-based eye-tracking setup, Fuchs reports on Polish HSs' use of grammatical gender cues. Unlike Spanish, where gender cues are frequent in definite articles, Polish cues appear on optional and infrequent adjectives. The results show that HSs can use gender on inflected adjectives to fixate on the target noun faster when the cue uniquely identifies it. This supports a grammatical account rather than probabilistic account of the facilitative use of grammatical gender, indicating that HSs access abstract syntactic information in real time to aid word recognition. Sagarra and Casillas add to the previous two eye-tracking studies by investigated factors (e.g., AoA, language proficiency and use) affecting Spanish stress-tense suffix associations among adult Spanish-English HSs, English-Spanish L2 learners, and Spanish monolinguals. Results showed that all groups were fixating on target verbs, with monolinguals displaying more fixations. Higher proficiency increased fixations in HSs and L2 learners, while increased use affected only HSs. The study highlighted HSs' reliance on lexical competitors and phonotactic frequency over token frequency or AoA. Altogether, the eye-tracking studies of Özsoy et al., Fuchs, and Sagarra and Casillas nicely showcase the importance of investigating HL from distinct linguistic domains (and language combinations) to complement each other and further expand our understanding of linguistic online processing in HL bilinguals.

The last two studies employed online methods that have only recently been used in psycholinguistic studies of bilingualism. Martohardjono et al. looked at pupillary responses to syntactic island constructions in two groups of Spanish/English bilinguals (HSs and late bilinguals). The findings offer insights into individual variation in language processing among HSs and late bilinguals, emphasizing the importance of considering usage patterns and exposure levels in assessing language competence. In the only neurolinguistic (EEG/ERP) study of this Research Topic, Luque et al. explored grammatical gender knowledge and processing among HSs and highlight the complex interplay between linguistic representations and processing mechanisms. More precisely, they showed that HSs' bilingual experience modulated some aspects of morphosyntactic processing (expressed as P600 and biphasic N400 effects), corroborating similar findings observed in the late L2 learners' literature (e.g., Alemán Bañón et al., 2018; Grey, 2023). These results highlight the necessity to further include brain methods in HL bilingualism in order to better understand what underlies HSs competence and processing outcomes.

Together, these studies provide a comprehensive overview of diverse heritage language linguistic phenomena, socio-cultural and (individual) processing/mechanistic aspects within HS communities, shedding new light on the multifaceted nature of bilingual language development and maintenance.

## Conclusion

This Research Topic offers an expansive overview of the intricate landscape of HL acquisition, processing, and maintenance. Through a diverse spectrum of empirical studies and theoretical explorations, the contributions within this volume have brought to light the dynamics underlying the development and usage of HLs across the lifespan. They further highlight the complexity and richness that underlies HL bilingualism, emphasizing the intricate interplay between linguistic, (neuro)cognitive and socio-cultural factors in shaping HL acquisition. The findings presented in this Research Topic serve as a steppingstone for future research and pedagogical innovations, advancing our understanding of HL phenomena and their implications for linguistic theory, language education, and societal multilingualism. Moving forward, it is essential that we embrace the complexities and uniqueness within HL bilingualism, aim for more precise and inclusive methodologies, acknowledging the diverse experiences and trajectories of HL speakers worldwide.

## Author contributions

FB: Conceptualization, Writing – original draft, Writing – review & editing. MK: Conceptualization, Writing – review & editing. SMPS: Conceptualization, Writing – review & editing.
